# Biomechanical effects of endplate sagittal coverage change on cervical disc replacement: a finite element analysis

**DOI:** 10.3389/fbioe.2024.1371548

**Published:** 2024-08-29

**Authors:** Lihua Chen, Haiyan Wang, Guangming Xu, Hao Liu

**Affiliations:** ^1^ Department of Rehabilitation, Shenzhen Bao’an Traditional Chinese Medicine Hospital, Guangzhou University of Chinese Medicine, Shenzhen, China; ^2^ Department of Famous Traditional Chinese Medicine Hall, Shenzhen Bao’an Chinese Medicine Hospital, Shenzhen, Guangdong, China; ^3^ Department of Orthopaedics, Shenzhen PingleOrthopedic Hospital and Shenzhen Pingshan Traditional Chinese Medicine Hospital, Shenzhen, Guangdong, China; ^4^ Department of Chinese Medical Master Hall, Ruikang Hospital affiliated to Guangxi University of Chinese Medicine, Nanning, Guangxi, China

**Keywords:** cervical vertebra, cervical disc replacement, endplate, finite element, heterotopic ossification

## Abstract

**Background:**

In recent years, the number of artificial cervical disc replacements has increased, and paravertebral ectopic ossification is a common complication. Although the exact mechanism is not clear, some studies suggest that it is related to the concentration of tissue stress caused by incomplete coverage of the trailing edge of the endplate. Therefore, this study performed a quantitative analysis to compare the biomechanical effects of different sagittal distances at the posterior edge of the endplate of the upper and lower prosthesis on the cervical spine and to explore the mechanical response of incomplete coverage of the posterior edge of the endplate on the paravertebral tissues.

**Methods:**

A C2-C7 nonlinear finite element model of the cervical spine was established and validated. Based on the cervical spine model, cervical disc replacement surgery models were constructed with different distances of sagittal distance at the posterior edge of the upper prosthetic endplate (0, 1, 2, 3 mm, respectively) and sagittal distance at the posterior edge of the lower prosthetic endplate (1, 2, 3 mm, respectively). Each model was subjected to the same 1Nm torque and 73.6N driven compressive load. Range of motion (ROM), intervertebral disc pressure (IDP), facet joint force (FJF), and endplate stress were measured at the cervical surgical and other segments.

**Results:**

Compared to the intact cervical spine model, the sagittal distance of the posterior edge of the prosthesis endplate at different distances increased the stress on the intervertebral disc and the capsular joint in the adjacent vertebral body segments to different degrees, especially in extension. In different directions of motion, the posterior margin sagittal distance of the posterior edge of the endplate of the lower prosthesis has a greater mechanical influence on the cervical spine compared to the posterior margin sagittal distance of the posterior edge of the endplate of the upper prosthesis. Compared with the intact model, the biomechanical parameters (ROM, FJF, endplate stress) of the C5-C6 segment increased the most when the sagittal distance of the posterior edge of the endplate of the upper prosthesis was 3 mm. Compared with the intact model, the maximum intervertebral disc stress of C4-C5 and C6-C7 was 0.57 MPa and 0.53 MPa, respectively, when the sagittal distance of the posterior edge of the upper prosthetic endplate was 3 mm.

**Conclusion:**

After the sagittal distance of the posterior edge of the prosthetic endplate was completely covered, the mechanical influence of the entire cervical spine was low. The sagittal distance at the posterior edge of the endplate of different sizes changed the motion pattern and load distribution of the implanted segment to some extent. When the sagittal distance between the prosthesis and the upper endplate was greater than or equal to 3 mm, the mechanical indices of the implanted segment increased significantly, increasing the risk of local tissue injury, especially during extension motion. Compared to the sagittal distance at the posterior edge of the endplate of the lower prosthesis, increasing the sagittal distance at the posterior edge of the endplate of the upper prosthesis has a greater effect on the mechanics of the cervical spine.

## 1 Introduction

Cervical disc replacement (CDR) is considered as an alternative to anterior cervical discectomy and fusion (ACDF) because it can effectively reduce the incidence of adjacent segment degenerative (ASD) changes and maintain the physiological curvature of the cervical spine (Phillips et al., 2015; Steinberger and Qureshi, 2020). CDR preserves the range of motion (ROM) of the operated segment while adequately decompressing the neural structure, thus realizing the design concept of “delaying the progression of degenerative changes in adjacent segments (Delamarter and Zigler, 2013; Jackson et al., 2016). However, with the accumulation of cervical artificial disc replacements and the gradual increase in postoperative follow-up, postoperative complications such as heterotopic ossification, kyphosis, and prosthesis displacement have occurred (Hui et al., 2021; Price et al., 2021). Previous literature reported that the incidence of heterotopic ossification (HO) after cervical artificial disc replacement was 17.8%–94.1% (Ganbat et al., 2016). The prosthesis type, poor prosthesis placement, and multisegmental replacement were associated with long-term HO (Yi et al., 2010).

The endplate insufficiency of the prosthesis is closely related to the occurrence of postoperative HO (Xu et al., 2021, Tu et al., 2012). The study showed (Shen et al., 2021) changes in endplate depth ratio and disc height are potential risk factors for HO after CDR. Periprosthetic HO reduces the range of motion and alters the biomechanical environment of the cervical spine, resulting in localized stress concentration (Yang et al., 2017). It is suggested that the mechanical concentration of the operative segment caused by incomplete endplate coverage is one of the main factors in the formation of HO (Xu et al., 2021). In addition, artificial cervical disc replacement increases the flexion and extension range of motion at the operative level, thereby altering the mechanical environment of the tissue. However, the sagittal distance at the posterior edge of the prosthesis endplate resulted in decreased endplate coverage, and the biomechanical relationship affecting the stability of the cervical spine has not been quantitatively analyzed.

Finite element analysis is a common method to analyze the biomechanical changes of the cervical spine and has been widely used in previous studies (Hua et al., 2020; Sun et al., 2022). Therefore, in this paper, the cervical finite element model of artificial disc replacement was established to investigate the mechanical effects of the coverage rate of the sagittal distance at the posterior edge of the endplate of different prostheses on cervical tissues. Then, we analyzed the range of motion (ROM) at different cervical segments, the intravertebral disc pressure (IDP) adjacent to the surgical segment, and the capsular stress at the surgical segment, as well as the endplate stress at the surgical segment.

## 2 Methods

### 2.1 Establishment of the cervical spine model

**FIGURE 1:**
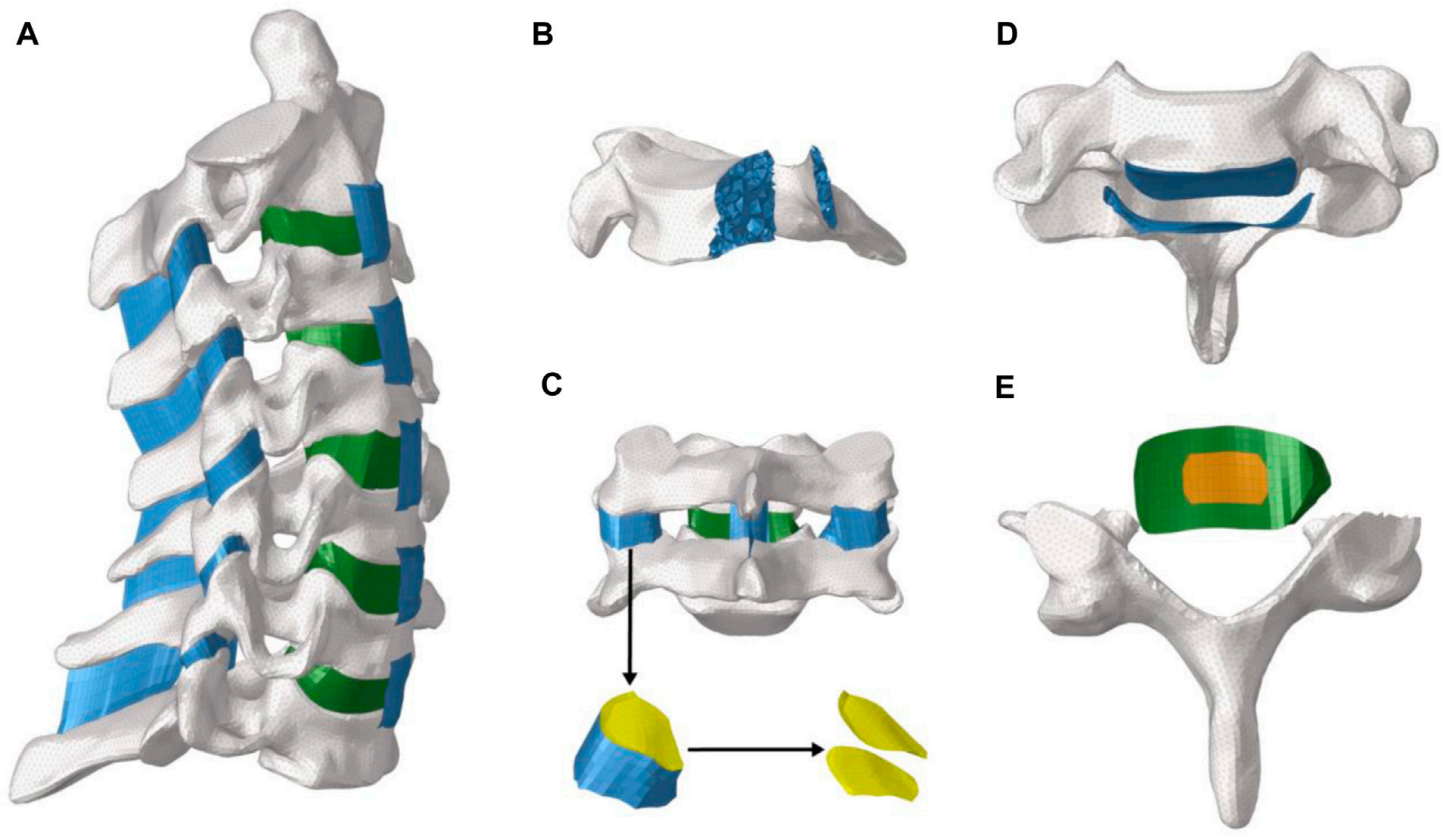
Overall and detailed view of the model. **(A)** whole view of the model; **(B)**. The internal structure of the vertebral body; **(C)**. Capsular ligaments of the vertebral body; **(D)**. Bony endplate; **(E)**. Intervertebral disc tissue and posterior bony structures.

A healthy male subject (age: 28 years; height: 171 cm; weight: 72 kg) computed tomography (CT) neck image was imported into Mimics 19.0, processed with image recognition, segmentation, and other functions, and a 3D solid model of the C2-C7 segments was extracted. We processed with surface fitting and other functions in the Geomagic studio. Then, the model was imported into Hypermesh for mesh division and material assignment. As shown in Figure 1, cortical bone, cancellous bone, intervertebral disc, joint capsule, bony endplate, joint capsule and ligament of the cervical vertebra were constructed in detail.

The materials of cortical bone, cancellous bone, and bony endplates were isotropic elastic (Wang et al., 2016; Purushothaman et al., 2021; Sun et al., 2022). The thickness of cortical bone was 1 mm (Zhang et al., 2019). The Materials of Mooney-Rivlin used for the nucleus pulposus and annulus fibrosus matrix (Schmidt et al., 2007; Sun et al., 2022). Orthotropic nonlinear elastic was used in the annulus of the intervertebral disc and Non-linear curves were used in the ligament tissue (Purushothaman et al., 2021). The material properties of cervical spine tissue are shown in Table 1.

**TABLE 1:** Material properties of cervical tissue.

Component	Element type	Material type	Material parameters	References
Cortical bone	Triangular shell	Isotropic elastic–plastic	*E* = 10.0 GPa *v* = 0.3	Sun et al. (2022)
Cancellous bones	Tetrahedral	Isotropic elastic-plastic	*E* = 300 MPa *v* = 0.3	Wang et al. (2016)
Bony endplate	Triangular shell	Isotropic elastic–plastic	*E* = 5.6 GPa *v* = 0.3	Purushothaman et al. (2021), Sun et al. (2022)
Nucleus pulpous	Hexahedral	Mooney-Rivlin	c10 = 0.12, c01 = 0.03	Schmidt et al. (2007), Sun et al. (2022)
Annulus fibrosus matrix	Hexahedral	Mooney-Rivlin	c10 = 0.18, c01 = 0.045	Schmidt et al. (2007), Sun et al. (2022)
Annulus fibrosus fibers	Quadrilateral membrane	Orthotropic nonlinear elastic	N/A	Purushothaman et al. (2021)
Ligaments	Q Quadrilateral membrane	Non-linear curves	N/A	Purushothaman et al. (2021)
Artificial cervical disc prosthesis				
Upper plate	Tetrahedral element		*E* = 210 GPa *v* = 0.3	Purushothaman et al. (2021)
Middle core	Tetrahedral element		*E* = 3 GPa *v* = 0.3	Purushothaman et al. (2021)
Lower plate	Tetrahedral element		*E* = 210 GPa *v* = 0.3	Purushothaman et al. (2021)

N/A: Not applicable.

### 2.2 Construction of CDR model

**FIGURE 2:**
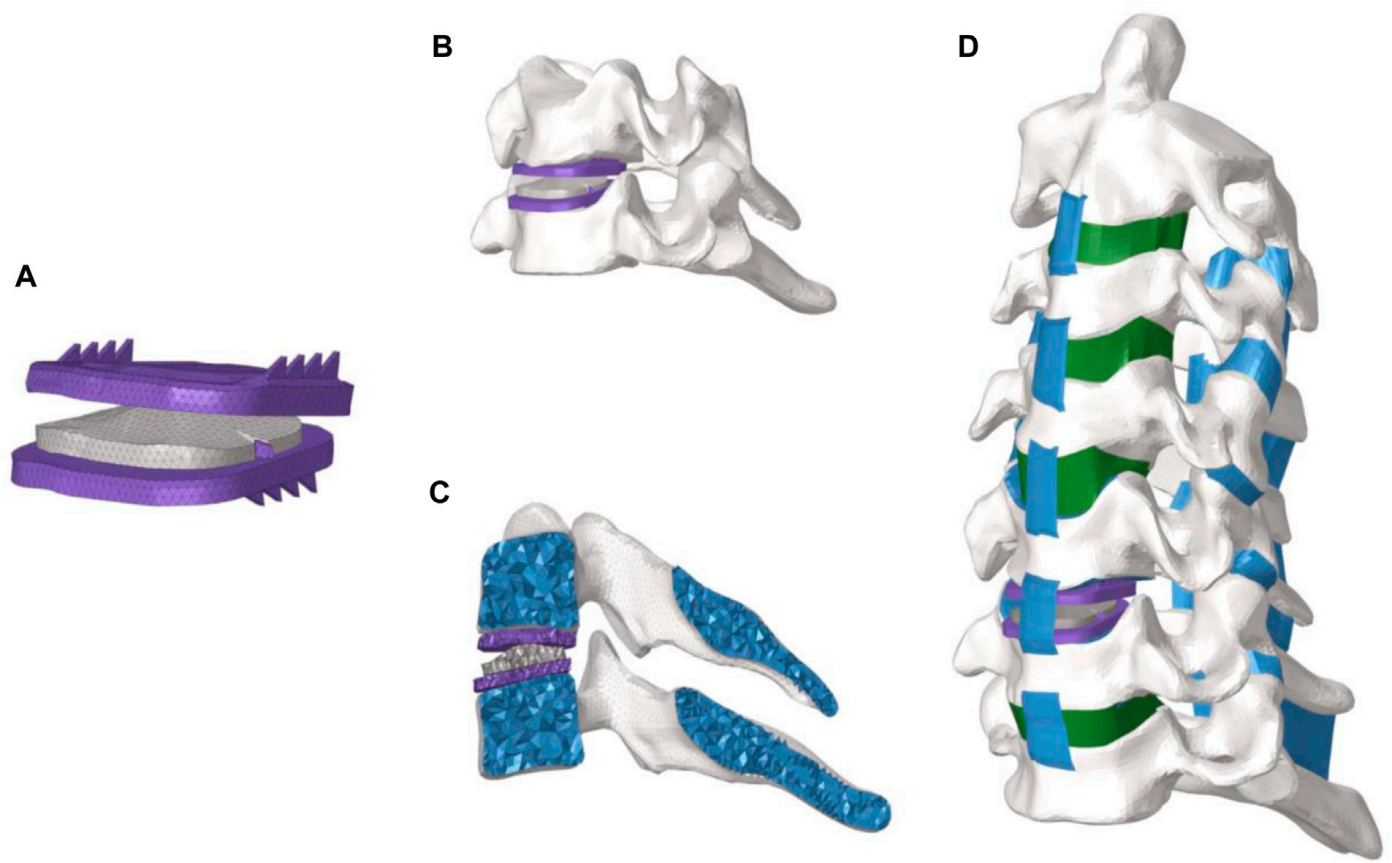
**(A)** View of the prosthesis for disc replacement; **(B)**. View of the disc prosthesis in the cervical spine segment; **(C)**. Internal view of the disc prosthesis in the cervical spine segment; **(D)**. Whole view of the disc prosthesis in the cervical spine.

The surgical model of cervical disc replacement is shown in Figure 2. To simulate the procedure of CDR, the C5-C6 disc was completely removed and the artificial disc was then inserted into the C5-C6 space. The interaction between the artificial disc and the endplate was set as a constraint. To investigate the mechanical difference of the sagittal distance at the posterior edge of the prosthesis endplate on the cervical spine tissue, we constructed the upper prosthesis endplate sagittal distance (UPESD-0) was 0 mm (UPESD-0), 1 mm (UPESD-1), 2 mm (UPESD-2), 3 mm (UPESD-3), and the lower prosthesis endplate sagittal distance was 1 mm (LPESD-1), 2 mm (LPESD-2), and 3 mm (LPESD-3).

### 2.3 Loads and boundary conditions

C7 was set as complete fixation, binding restraint between prosthesis and bone, and frictionless contact between joint capsules. In all cervical FE models, loads were applied to the upper surface of C2 and the lower surface of C7 was completely fixed. To simulate the mass and muscle activity of the adult skull under physiological conditions, an axial load of 73.6N was applied to the center of C2 and a torque load of 1 Nm was applied to simulate flexion, extension, lateral bending, and rotation of the cervical spine (Sun et al., 2023).

## 3 Results

### 3.1 Model validation

**FIGURE 3:**
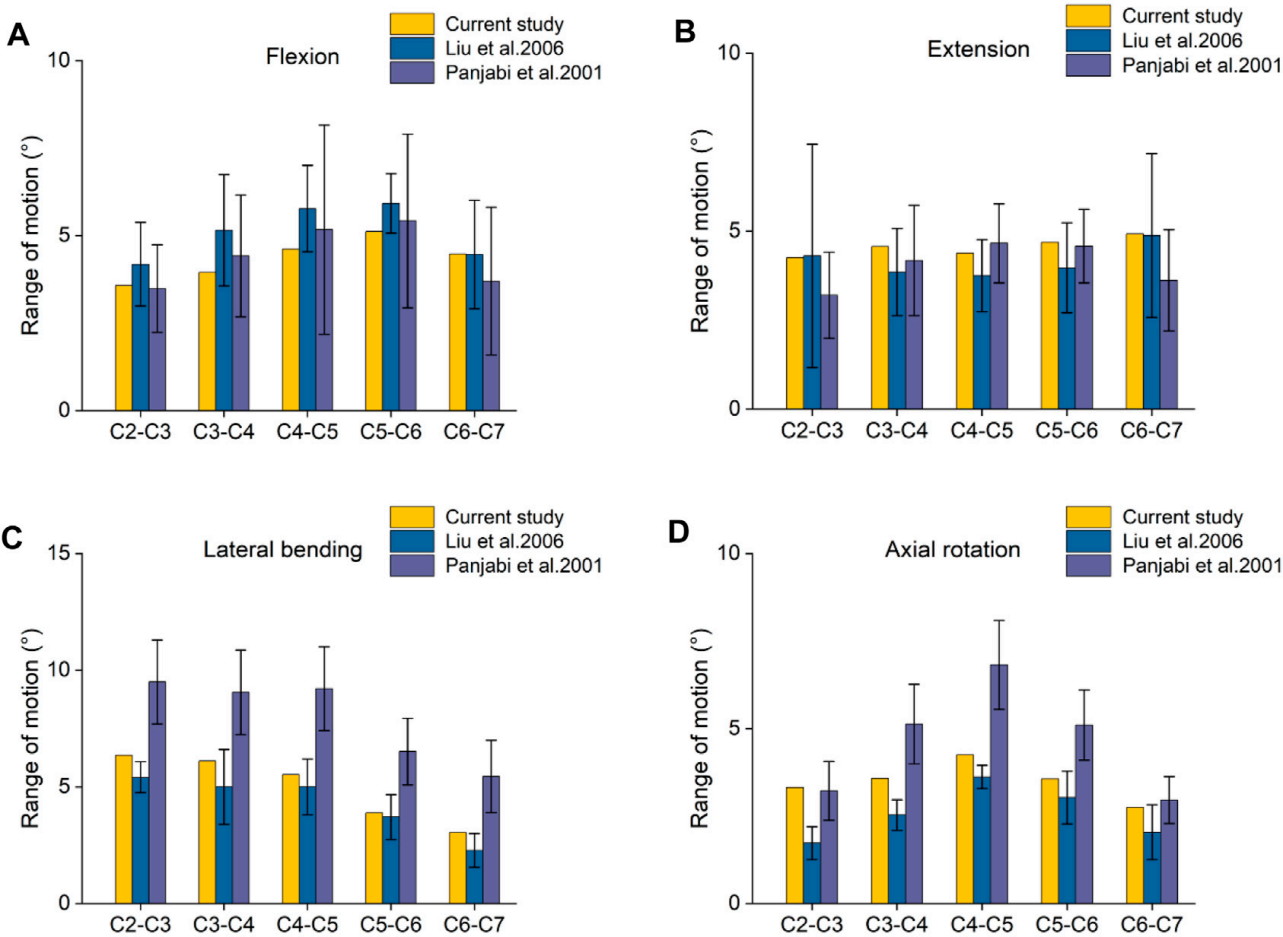
Verification of range of motion in the cervical spine model. **(A)**. The ROM value of flexion motion; **(B)**. The ROM value of the extension motion; **(C)**. The ROM value of lateral bending; **(D)**. The ROM value of axial rotation.

To verify the validity of the model, the biomechanical properties of the cervical spine during forward flexion, backward extension, left and right lateral bending, left and right rotation and compression were simulated, and compared with the experimental data of Liu et al. (2016) and Panjabi et al. (2001). The data of intervertebral relative motion calculated in this study are consistent with the results of previous studies in terms of trend and value in Figure 3, which indicates the reliability of the model prediction.

### 3.2 Intersegmental ROM

**FIGURE 4:**
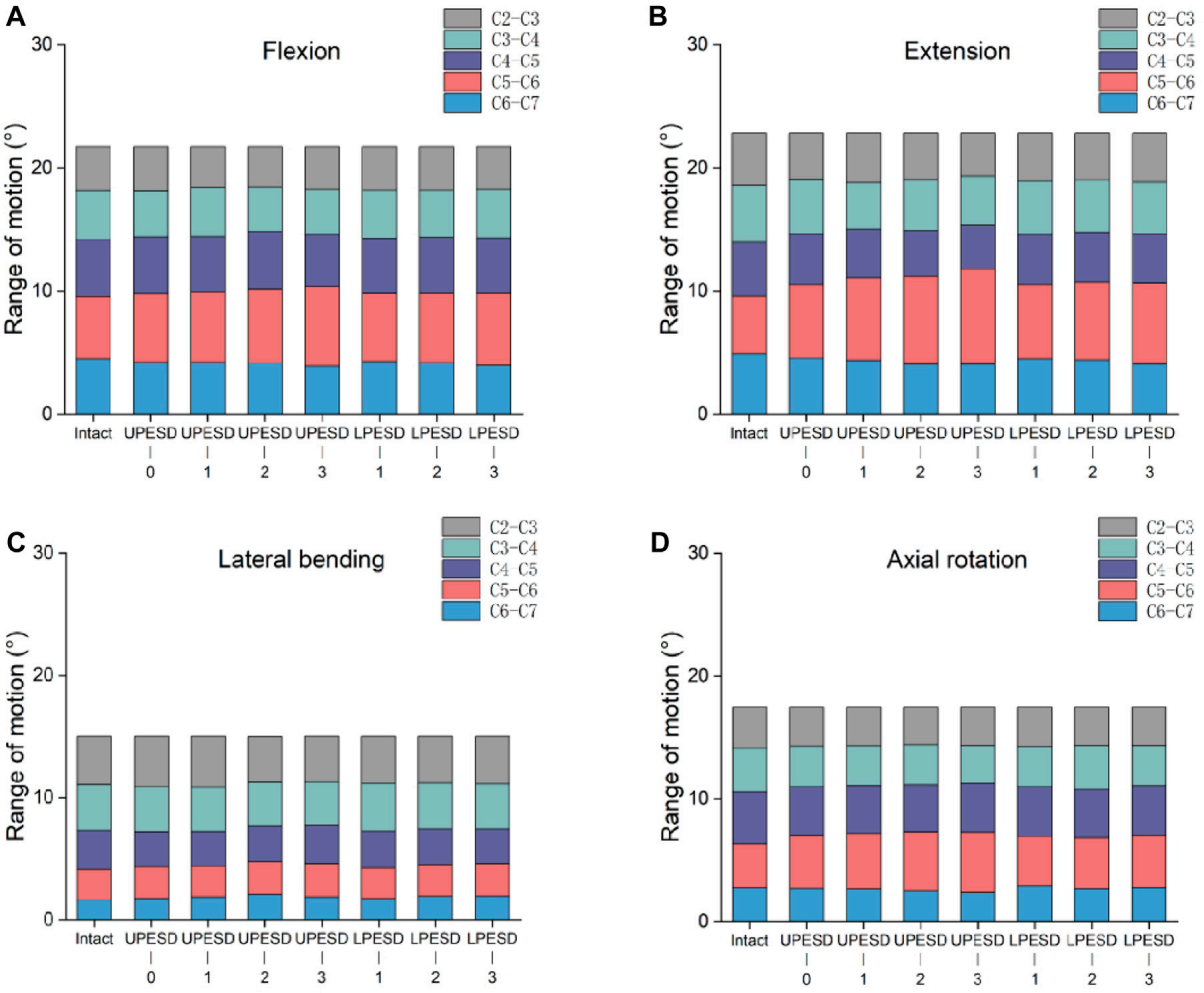
Cervical segmental range of motion values in the complete model and the surgical model. **(A)**. The ROM values of different segments in flexion motion; **(B)**. The ROM values of different segments in the extension motion; **(C)**. The ROM values of different segments with lateral bending. **(D)**. The ROM values of different segments under axial rotation.

Intersegmental changes to the same range of motion before surgery were compared in each group of fixed models with different prostheses inserted into the cervical spine at a sagittal distance from the posterior edge of the endplate in Figure 4. In the flexion condition, the motion of the sagittal distance at the posterior edge of the prosthesis endplate in ROM segment C5 -C6 was greater than that at the posterior edge of the prosthesis endplate, which was 3%, 6%, and 6%, respectively. In the posterior extension condition, the value of the prosthesis model with complete coverage of the endplate increased by 27% compared with the normal model and gradually increased with the increase of the sagittal distance between the prosthesis and the posterior edge, especially in the three groups with the sagittal distance of the posterior edge of the upper prosthesis, which increased by 42%, 52%, 64%, respectively. Compared with the sagittal distance of the posterior edge of the endplate of the lower prosthesis, the range of motion of the sagittal distance of the posterior edge of the endplate of the upper prosthesis increased by 10%,13%, and 17%, respectively. Under rotating conditions, the motion of C5- C6 segments increased, while most of the other segments decreased.

### 3.3 IDPs adjacent to surgical segments

**FIGURE 5:**
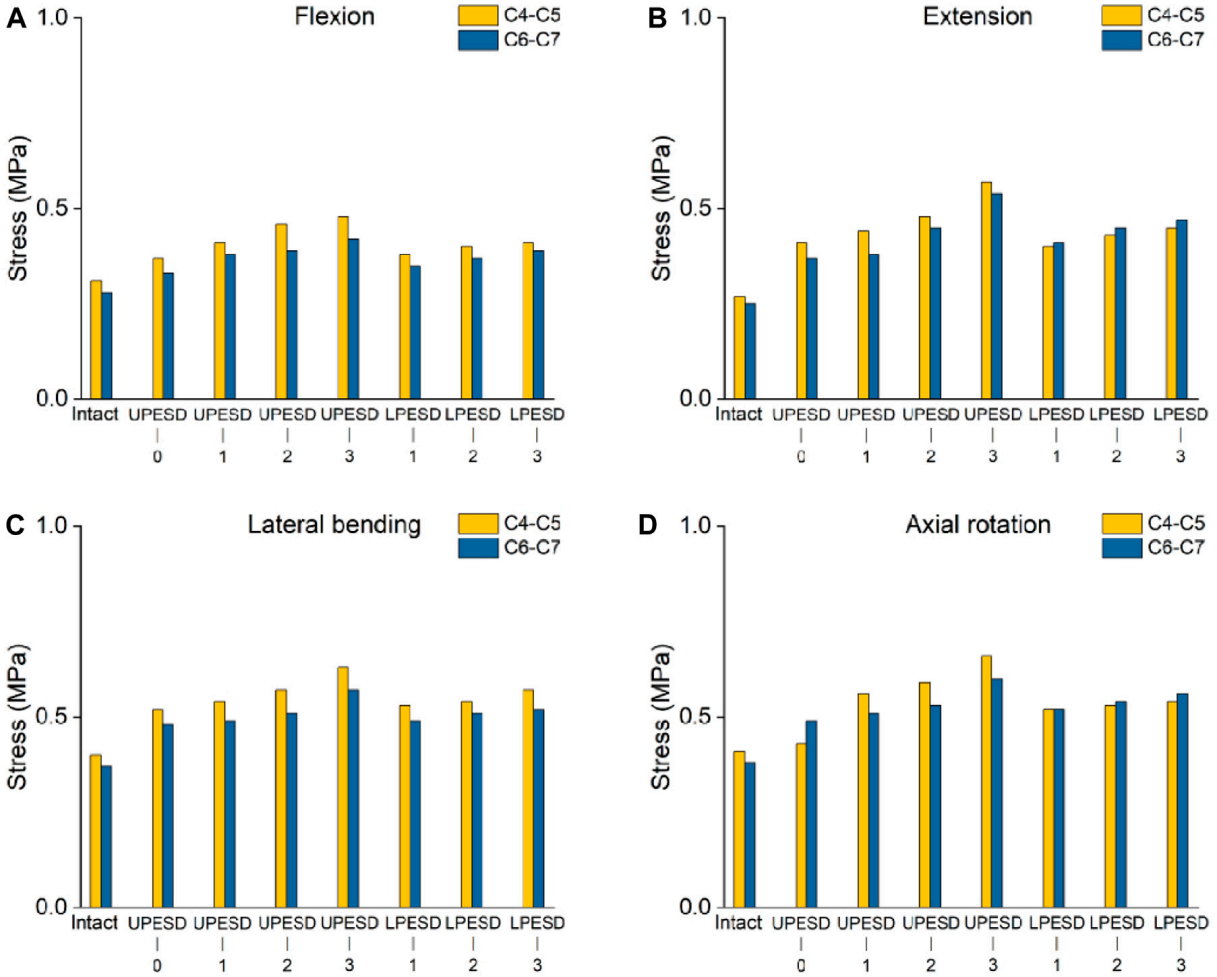
Stress values of the intervertebral discs adjacent to the operative segment of the complete model and the operative model. **(A)**. The stress value of the intervertebral disc in the adjacent segments of the operation in different models of flexion motion. **(B)**. The stress value of the intervertebral disc in the adjacent segments of the operation in different models with extension motion. **(C)**. The stress value of the intervertebral disc in the adjacent segments of the operation in different models with lateral bending. **(D)**. The stress value of the intervertebral disc in the adjacent segments of the operation in different models of axial rotation activity.

The calculation results of the maximum equivalent stress of the intervertebral disc under four working conditions are shown in Figure 5. In the four conditions, the maximum stress values of C4-C5 and C6-C7 intervertebral discs were slightly increased when the prosthesis completely covered the sagittal distance of the endplate compared with the complete model. The sagittal distance of the posterior edge of the prosthesis endplate increased to varying degrees, and the most obvious increase was found in the extension condition. In the posterior extension condition, compared with the complete model, when the sagittal distance of the posterior edge of the upper and lower endplates of the prosthesis was 1, 2, 3 mm, C4-C5 increased by 63%, 76%, 111%, 25%, 59%, 67% respectively, while the corresponding C6-C7 increased by 52%, 66%, 92%, 51%, 63%, 74%, respectively. However, the maximum stresses in the two segments were 0.57 MPa and 0.53 MPa, respectively.

### 3.4 Stress of endplate in C5-C6 segments

**FIGURE 6:**
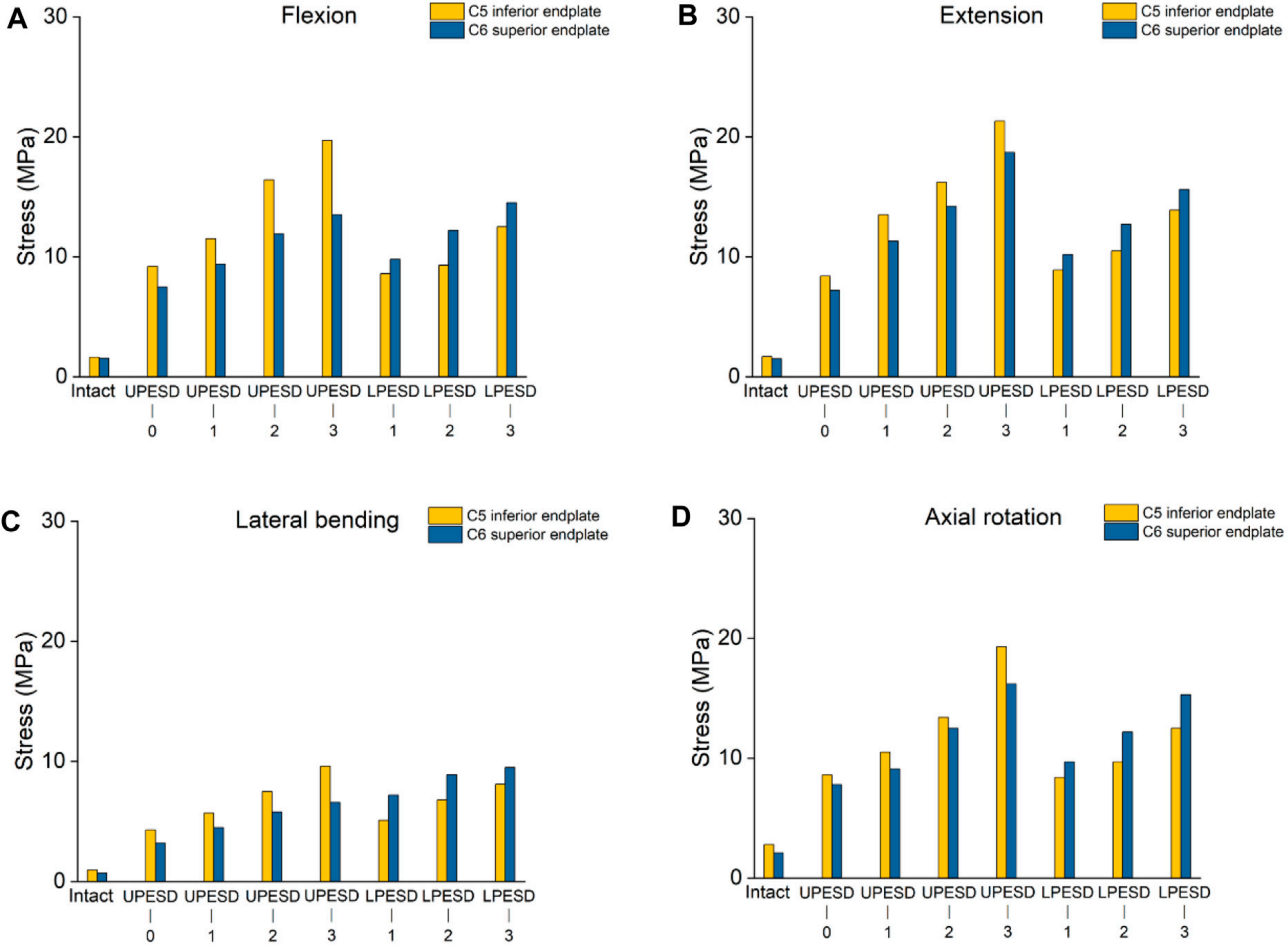
Stress values of the osseous endplate at the operative segment of the complete model and the operative model. **(A)**. The stress value of the bony endplate at the operative segment in different models of flexion motion. **(B)**. The stress value of the bony endplate at the operative segment in different models with extension motion; **(C)**. The stress value of the bony endplate at the operative segment in different models with lateral bending. **(D)**. The stress value of the bony endplate at the operative segment in different models of axial rotation activity.

Under the four conditions, the stress of the upper and lower endplates increased significantly after the replacement in Figure 6. Compared with the complete model, the maximum stress values of the upper and lower endplates increased. When the sagittal distance of the posterior edge of the upper prosthesis endplate changes, the stress value of the endplate under C5 is higher than that on C6. However, in the posterior sagittal distance model of the lower prosthesis endplate, contrary to the above results, the stress value of the upper endplate on C6 is higher than that of the endplate under C5.

### 3.5 Capsular stress of FJC

**FIGURE 7:**
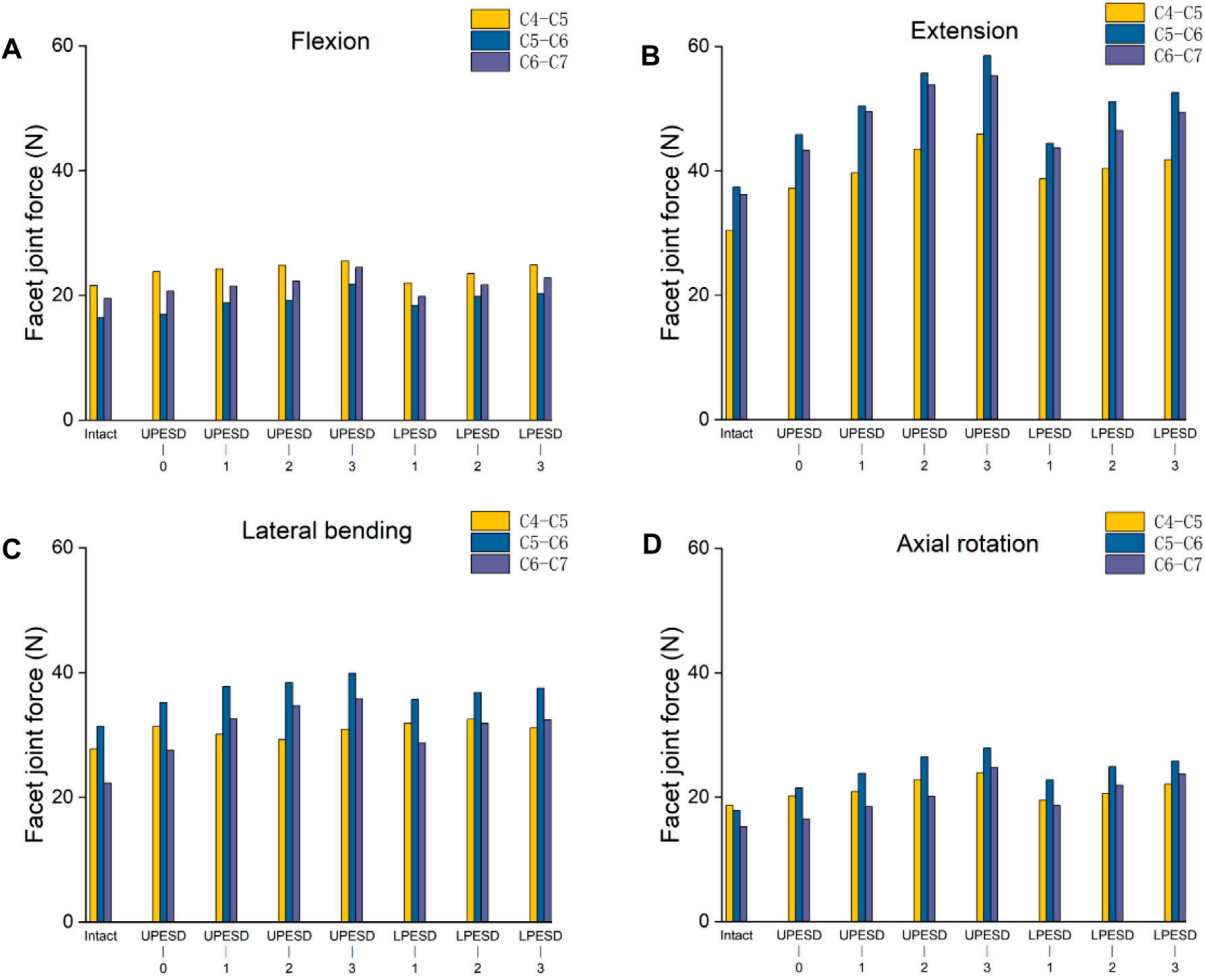
Stress values of the joint capsule at the operative segment and adjacent segment of the complete model and the surgical model. **(A)**. The stress value of the joint capsule at the operative segment and adjacent segment in different models of flexion. **(B)**. The stress value of the joint capsule at the operative segment and the adjacent segment in different models with extension. **(C)**. The stress value of the joint capsule at the operative segment and adjacent segment in different models with lateral bending. **(D)**. The stress values of the joint capsule at the operative segment and adjacent segment in different models of axial rotation activity.

Under flexion, lateral flexion, and rotation conditions in Figure 7, the capsular stress of the operative segment and the adjacent segment showed an irregular trend of change, and the range of increase or decrease was small. However, in the posterior extension condition, the stress of the facet joint capsule in the operative segment and the adjacent segment increased significantly. Compared with the complete model, the capsular stress at C4-C5 levels increased by 22%, 30%, 43%, 51%, 27%, 33%, 38%, C5-C6 segments increased by 22%, 38%, 49%, 56%, 18%, 37%, 41%, and C6-C7 segments 19%, 36%, 42%, 53%, 21%, 38%, 36%. According to the above results, compared with the variation of the sagittal distance at the posterior edge of the end plate of the lower prosthesis, the stress increased by the sagittal distance at the posterior edge of the end plate of the upper prosthesis was more obvious.

## 4 Discussion

In this study, a finite element model of the cervical spine was constructed and the validity of the model was verified by analyzing the ROM values of the cervical spine. The current study used the cervical spine model to compare the mechanical indexes of different sagittal distances at the posterior edge of the upper and lower prosthesis endplates and to explore the effects of different sagittal distances at the posterior edge of the prosthesis endplates on the cervical biomechanics.

CDR preserves the range of motion at the operative level compared to anterior cervical fusion, thereby preventing degeneration of adjacent levels. However, further studies (DiAngelo et al., 2004; Bertagnoli et al., 2005; Patwardhan and Havey, 2020) showed that CDR increased the range of flexion and extension motion compared to the preoperative range of motion. Our results showed that different endplate coverage increased the mechanical indexes of adjacent segments at the C5-C6 surgical segment. During extension, the ROM value of the surgical segment increased by a maximum of 64% when the sagittal distance of the posterior margin of the upper prosthesis endplate was 3 mm, compared with that of the normal model. The use of artificial disc prostheses changes the angle of extension and the pattern of movement, and the increased range of motion at the segmental level increases the pressure on the uncinate and facet joints (Lazaro et al., 2010). This may be due to the postoperative instability of the cervical spine in the posterior extension position after the anterior ligament and annulus fibrosus were removed during CDR. In addition, the upper endplate model showed a greater increase in motion compared to the lower endplate model. The insufficient sagittal distance coverage area at the posterior edge of the endplate may directly affect the stability of the cervical spine.

Changes in the biomechanical environment of the surgical segment caused by artificial disc implantation led to local stress concentration in the surgical segment, but have relatively little effect on adjacent segments. HO is often found in surgical segments, and stress concentration is one of the main causes (Hui et al., 2021). In this study, with the increase of sagittal distance at the posterior edge of the prosthesis endplate, the stress of the intervertebral disc and joint capsule in the adjacent segments of C5-C6 increased. The stress of the endplate increased significantly at C5-C6 segments, especially when the stress was greater than 3 mm. However, compared with the sagittal distance of the posterior edge of the endplate of the lower prosthesis, the stress increased by the sagittal distance of the posterior edge of the endplate of the upper prosthesis was more obvious, and the concentration of stress would increase the occurrence of ectopic ossification and affect the stability of the cervical spine. Clinical research (Xu et al., 2021) showed that HO after CDR was more likely to occur when the error distance between the prosthesis and the sagittal plane of the endplate was greater than 2.5 mm, which had a good agreement with our results. HO can lead to decreased stability of the cervical spine (Jin et al., 2013). Due to the changes in the biomechanical environment of the surgical segment and the endplate, the long-term sustained loading of the vertebrae in the replacement segment will inevitably lead to injury or degradation of the segment, thus accelerating the formation of ossification.

If the implant is improperly placed or undersized, high contact stresses will occur at the endplate interface, and high implant-bone interface stresses may affect the stability of the implant and the cervical segment (Lin et al., 2009). An artificial disc prosthesis based on the physiological curvature of the endplate can reduce the stress at the prosthesis-bone interface (Yu et al., 2016). Therefore, better endplate coverage may better maintain prosthesis and segment stability and reduce stress concentration. Clinical studies have shown that incomplete endplate coverage is an important cause of ectopic ossification. Combined with our findings above, lack of endplate coverage increases stress on the tissue surrounding the replacement segment and alters its motion pattern. Previous studies have indicated that the structural integrity of the endplate should be assessed preoperatively and that the prosthesis should cover the endplate as much as possible to distribute the load evenly rather than in concentrated areas. Through intraoperative manipulation, the surgeon prevents the prosthesis from being placed too far posteriorly to compress the spinal cord, which inevitably results in incomplete coverage of the posterior margin (Choi et al., 2020). Therefore, intraoperative positioning should be enhanced to better cover the posterior edge of the endplate and distribute stress as evenly as possible (Goel et al., 2012).

The current model has some inherent limitations and simplifications. First, the model lacks the spinal cord, blood vessels, muscles, and other tissues, and further improvement is needed to establish a more detailed model. Second, the flexion, extension, lateral flexion and other movements simulated in this paper are passive movements. However, in the human body, they are active muscle movements. In the future, they should be combined with active muscle forces to simulate the mechanical loading mode of the human body. Third, because there are many types of artificial intervertebral discs, only one of them is studied in this paper, and more sample studies are needed in the future.

## 5 Conclusion

In this study, a three-dimensional nonlinear finite element model of the cervical spine was established to verify the validity of the model. Based on this model, the effects of sagittal distance of different prosthesis endplates on the biomechanics of the cervical spine were analyzed. The results showed that complete coverage of the posterior margin of the prosthesis endplate had the least effect on the mechanical indexes of the cervical spine. However, with the increase of incomplete coverage of the posterior margin of the prosthesis endplate, the mechanical indexes of the implanted segments were significantly increased, which increased the risk of local tissue injury and ectopic ossification. Compared with the prosthesis-inferior endplate sagittal distance ratio, the prosthesis-superior endplate sagittal distance ratio has a greater effect on the mechanics of the cervical spine. These conclusions provide an important reference for the placement of intraoperative prostheses and the subsequent improvement of prostheses.

## Data Availability

The original contributions presented in the study are included in the article/Supplementary Material, further inquiries can be directed to the corresponding author.
